# Indirect comparison of glucagon like peptide-1 receptor agonists regarding cardiovascular safety and mortality in patients with type 2 diabetes mellitus: network meta-analysis

**DOI:** 10.1186/s12933-020-01070-z

**Published:** 2020-06-22

**Authors:** Osamah M. Alfayez, Omar A. Almohammed, Omar S. Alkhezi, Abdulaali R. Almutairi, Majed S. Al Yami

**Affiliations:** 1 Department of Pharmacy Practice, College of Pharmacy, Qassim University, Qassim, Saudi Arabia; 2 Department of Clinical Pharmacy, College of Pharmacy, King Saud University, Riyadh, Saudi Arabia; 3 Saudi Food and Drug Authority, Riyadh, Saudi Arabia; 4 Department of Pharmacy Practice, College of Pharmacy, King Saud bin Abdulaziz University for Health Sciences, Riyadh, Saudi Arabia

**Keywords:** Cardiovascular disease, Diabetes mellitus, Type 2, Meta-analysis, Myocardial infarction, Stroke, Heart failure

## Abstract

**Background:**

The cardiovascular outcome trials (CVOTs) have shown that glucagon like peptide-1 receptor agonists (GLP1RAs) have varying degrees of cardiovascular (CV) safety in patients with type 2 diabetes mellitus (T2DM.) The lack of any head-to-head comparative trials among GLP1RAs urged the need for an indirect comparison of these agents. Therefore, this study was conducted to indirectly compare the CV safety and mortality effects among different GLP1RAs in patients with T2DM using network meta-analysis (NMA).

**Methods:**

Medline was searched to identify GLP1RA CVOTs to date. The outcomes of interest were CV death, myocardial infarction (IM), stroke, and death from any cause. An NMA with binomial likelihood logit link model was used for the binary outcomes. We conducted both fixed effects and random effects models for each outcome, and selected the best model based on the deviance information and the average posterior residual deviance. This NMA was reported in accordance with the preferred reporting items for systematic reviews and meta-analyses (PRISMA-NMA).

**Results:**

A total of seven GLP1RA CVOTs were included having 56,004 patients. The NMA results showed that oral semaglutide was statistically better than exenatide (OR 0.47, 95% CI 0.21–0.99), dulaglutide (OR 0.46, 95% CI 0.20–0.97), albiglutide (OR 0.45, 95% CI 0.19–0.97), lixisenatide (OR 0.43, 95% CI 0.19–0.92) in reducing CV death events. No significant differences were detected between most of the treatments regarding reducing death from any cause, MI and stroke events. The ranking results showed that oral semaglutide had the highest probability to be ranked first (> 90%) in reducing CV death and death from any cause. Moreover, once weekly semaglutide had the highest probability to be ranked first in reducing MI and stroke events.

**Conclusion:**

The GLP1RAs have shown significant benefits in terms of CV safety. The indirect comparison and ranking probability results have shown that one weekly semaglutide and oral semaglutide seems to be the preferred option in patients with T2DM and established or at high risk of CVD. This result can aid health care providers, pharmacy and therapeutics committees in hospitals, and insurance companies when deciding which GLP1RA to start or add to their formulary.

## Background

Diabetes mellitus is a major health condition that affects more than 382 million adults worldwide, and this number is expected to reach 592 million by 2035 [1]. Patients with diabetes are at a higher risk of developing cardiovascular (CV) complications, which can be fatal. Cardioprotective effects of some antidiabetic drugs, along with the improvement in the provided care, has led to the reduction in the rate of CV events over the years. However, the rate of the CV events remains significantly higher in diabetic patients than in the general population [2].

Glucagon like peptide-1 receptor agonist (GLP1RA) based therapies have been in use since 2005 and GLP1RAs have been extensively studied for the treatment of type 2 diabetes mellitus (T2DM). Most of the GLP1RAs, including lixisenatide (short-acting) and dulaglutide (long-acting), are administered as a subcutaneous injection either once daily or weekly [3]. Recently, the first oral formulation of a GLP1RA, daily oral semaglutide, was approved by the US food and drug administration (FDA) [3]. GLP1RAs are classified based on their structure as either exendin-4 based (e.g. lixisenatide and exenatide) or human GLP-1 analogue (e.g. liraglutide, dulaglutide, semaglutide and albiglutide) [3, 4]. GLP1RAs act by increasing insulin secretion, decreasing glucagon secretion, and improving satiety. They have also been shown to have efficacy in reducing body weight and have potential antiatherothrombotic effects [3, 5].

In 2008, the US FDA mandated that all novel antidiabetic drugs must be evaluated by manufacturers to assess their CV safety profile [6]. Since then, several GLP1RA cardiovascular outcome trials (CVOTs) have been conducted. To date, there are a total of seven GLP1RA CVOTs published [7–13], and one trial is ongoing [14]. The trials have shown that GLP1RAs have varying degrees of CV safety and recent meta-analyses have shown that this class has a promising CV safety profile [15–19]. However, it is still not clear which GLP1RA is preferred for CV safety. Furthermore, there is a lack of any head-to-head comparative trials among GLP1RAs. Therefore, this study was conducted to indirectly compare the CV safety and mortality effects among different GLP1RAs in patients with T2DM using network meta-analysis (NMA).

## Methods

### Literature search, selection criteria and risk of bias

PubMed was searched from inception to November 2019 with the aim to identify GLP1RA CVOTs. Published randomized double blind placebo-controlled studies that have addressed the CV safety of GLP1RA as their primary outcomes were targeted. The outcomes of interest were major adverse cardiovascular events (MACE; defined as the composite endpoint of CV mortality, nonfatal myocardial infarction (MI) and nonfatal stroke termed three-point MACE). In addition to MACE, death from any cause, stroke, nonfatal stroke, MI, nonfatal MI and hospitalizations for heart failure (HF) outcomes were included. The search terms used were “glucagon like peptide-1,” “GLP-1 receptor agonist,” “liraglutide,” “semaglutide,” “albiglutide,” “exenatide,” “stroke,” “nonfatal stroke,” “myocardial infarction,” “nonfatal myocardial infarction,” “heart failure,” “CV death,” and “CV mortality.” Results were further limited to randomized controlled trials and English language. Published data were extracted from the published studies by two authors (OMA and OAA) independently, and reviewed by a third one (OSA). The risk of bias was assessed using the Cochrane risk of bias assessment tool [20].

### Data synthesis and analysis

A Bayesian NMA, using binomial likelihood with logit link model and non-informative prior distribution, was used for the analysis of dichotomous outcomes. Fixed and random effects models were conducted for each outcome, then the most suitable model based on the deviance information and the average posterior residual deviance was selected. We ran inconsistency models to assess the consistency using the residuals in inconsistency plots. Analysis was conducted using WinBUGS 1.4.3 (MRC Biostatistics Unit; Cambridge, United Kingdom) through NetMetaXL 1.6.1 (Canadian Agency for Drugs and Technologies in Health; Ottawa, Canada) [21]. We evaluated the possible covariates that might not be similar between the trials using mate-regression analyses which were performed with GeMTC GUI package [22]. The NMA was reported according to the statement of preferred reporting items for systematic reviews and meta-analyses involving network meta-analysis (PRISMA-NMA) in Additional file 1: Table S1 [23].

## Results

### Search results and study characteristics

A total of 79 studies were identified in the systematic search, and after title and abstract screening a total of seven GLP1RA CVOTs were included, the flowchart of included and excluded studies was illustrated in Additional file 2: Figure S1. The included seven studies were as follow, ELIXA (lixisenatide), LEADER (liraglutide), SUSTAIN-6 (semaglutide), EXSCEL (exenatide), Harmony outcomes (albiglutide), REWIND (dulaglutide) and PIONEER-6 (oral semaglutide) [7–13]. All the studies have compared the CV safety of one of the GLP1RAs to placebo, both as added on therapy to the standard of care, as shown in Table 1. The PIONEER-6 trial was the only trial that included an oral formulation that was administered once daily (semaglutide), whereas all the remaining trials included injectable formulations. In ELIXA and LEADER trials, the drugs were administered once daily (i.e. lixisenatide and liraglutide) and in the rest, once weekly (i.e. semaglutide, albiglutide, exenatide and dulaglutide).

**Table 1:** Comparison of glucagon like peptide-1 receptor agonist (GLP1RA) cardiovascular outcome trials (CVOTs)

Study	Design	Medications	Sample size N	Male sex N (%)	Age, years Mean (SD)	Median follow-up years	Duration of diabetes, years Mean (SD)	HbA1c, % Mean (SD)	Existence of CVD N (%)	Statin use N (%)	SGLT-2i use N (%)
ELIXA	Randomized, double-blind, placebo-controlled trial	Lixisenatide once daily vs placebo	6068	4207 (69)	59.9 (9.7)^b^	2.1	9.2 (8.2)^b^	7.7 (1.3)^b^	6068 (100)	5627 (92.7)	NA
LEADER	Randomized, double-blind, placebo-controlled trial	Liraglutide once daily vs placebo	9340	6003 (64)	64.2 (7.2)^b^	3.8	12.8 (8.0)^b^	8.7 (1.6)^b^	7598 (81)	6741 (72.2)	NA
SUSTAIN-6	Randomized, double-blind, placebo-controlled trial	Semaglutide once weekly vs placebo	3297	2002 (61)	64.6 (7.4)	2.1	13.9 (8.1)	8.7 (1.5)	2735 (83)^c^	2399 (72.8)	5 (0.2)
EXSCEL	Randomized, double-blind, placebo-controlled trial	Exenatide once weekly vs placebo	14,752	9148 (62)	62.7 (3.6)^d^	3.2	12.3 (3.2)^d^	8.1 (0.5)^d^	10,782 (73)	10,836 (73.5)	77 (0.9)
HARMONY	Randomized, double-blind, placebo-controlled trial	Albiglutide once weekly vs placebo	9463	6569 (69)	64.1 (8.7)^b^	1.6	14.1 (8.6)^b^	8.7 (1.5)^b^	6678 (71)	7955 (84.1)	575 (6.1)
REWIND	Randomized, double-blind, placebo-controlled trial	Dulaglutide once weekly vs placebo	9901	5312 (53.7)	66.2 (6.5)	5.4	10.5 (7.3)^b^	7.3 (1.1)^b^	3114 (31)	6547 (66.1)	3 (0.0)
PIONEER-6	Randomized, double-blind, placebo-controlled trial	Semaglutide once daily^a^ vs placebo	3183	2176 (68.4)	66 (7.0)	1.3	14.9 (8.5)	8.2 (1.6)	2695 (85)^c^	2712 (85.2)^e^	305 (9.6)

*ELIXA* the evaluation of lixisenatide in acute coronary syndrome trial; *LEADER* the liraglutide effect and action in diabetes evaluation of cardiovascular outcome results trial; *SUSTAIN-6* the preapproval trial to evaluate cardiovascular and other long-term outcomes with semaglutide in subjects with type 2 diabetes; *EXSCEL* the exenatide study of cardiovascular event lowering; *HARMONY* a long term, randomized, double blind, placebo-controlled study to determine the effect of albiglutide, when added to standard blood glucose lowering therapies, on major cardiovascular events in patients with type 2 diabetes mellitus; *CVD* cardiovascular disease; *SD* standard deviation; *HbA1c* glycated hemoglobin

^a^Oral semaglutide

^b^Data retrieved from the treatment arm

^c^Presented in the trial as total of CVD and/or chronic kidney disease

^d^Estimated based on the median range, and the sample size

^e^Presented in the trial as lipid lowering agents in general

The studies included a total of 56,004 patients with T2DM who had either established CVD or risk factors for CVD. The sample size in the trials ranged from 3183 patients in the PIONEER-6 trial to 14,752 patients in the EXSCEL trial. The mean age of participants ranged from 59.9 to 66.2 years, with the REWIND trial including older patients. The median follow-up ranged from 1.3 years (PIONEER-6) to 5.4 years (REWIND). The ELIXA trial included only patients with recent acute coronary syndrome. Whereas the remaining trials included varying percentages of patients with established CVD, for example 31% of patients in the REWIND trial had established CVD. The use of sodium glucose cotransporter-2 inhibitors (SGLT2is) varied among the trials; the Harmony outcomes and PIONEER-6 trials had the highest use. Lastly, the use of statins ranged from 66 to 92% of patients in the included trials (Table 1). The network plot is presented in Additional file 3: Figure S2, the risk of bias assessment showed that all the included trials had a low risk of bias as presented in Additional file 4: Table S2, and there were no signs of inconsistency between the trials in the inconsistency plots for fixed models as demonstrated in Additional file 5: Figure S3.

### MACE

**Fig. 1:**
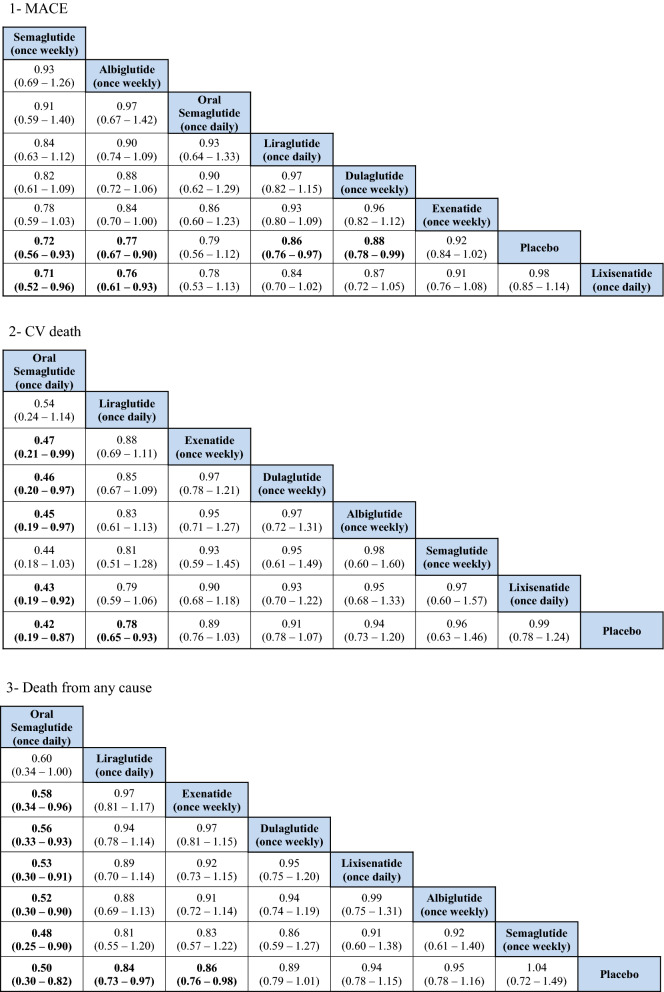

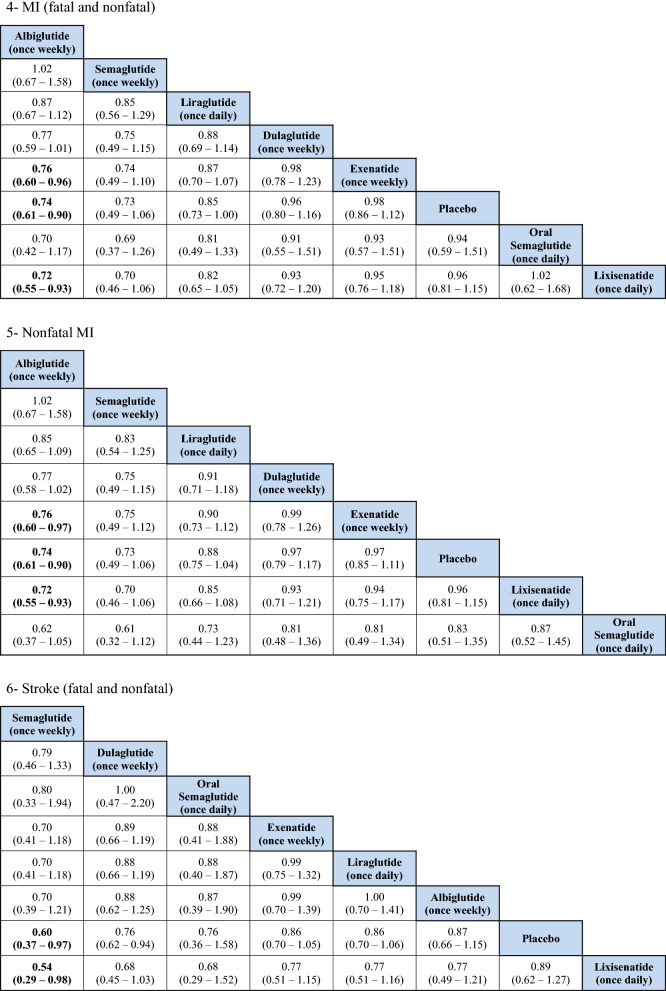

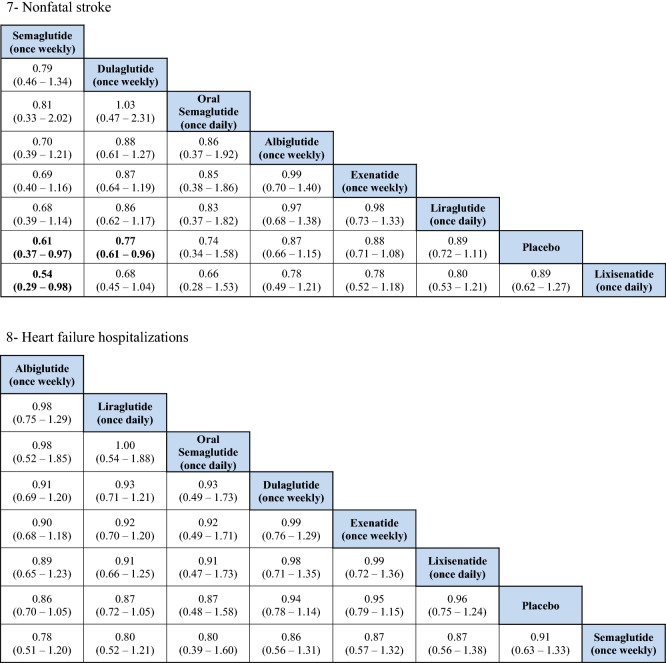
Network meta-analysis results of GLP1RAs and placebo using fixed effect model

**Fig. 2:**
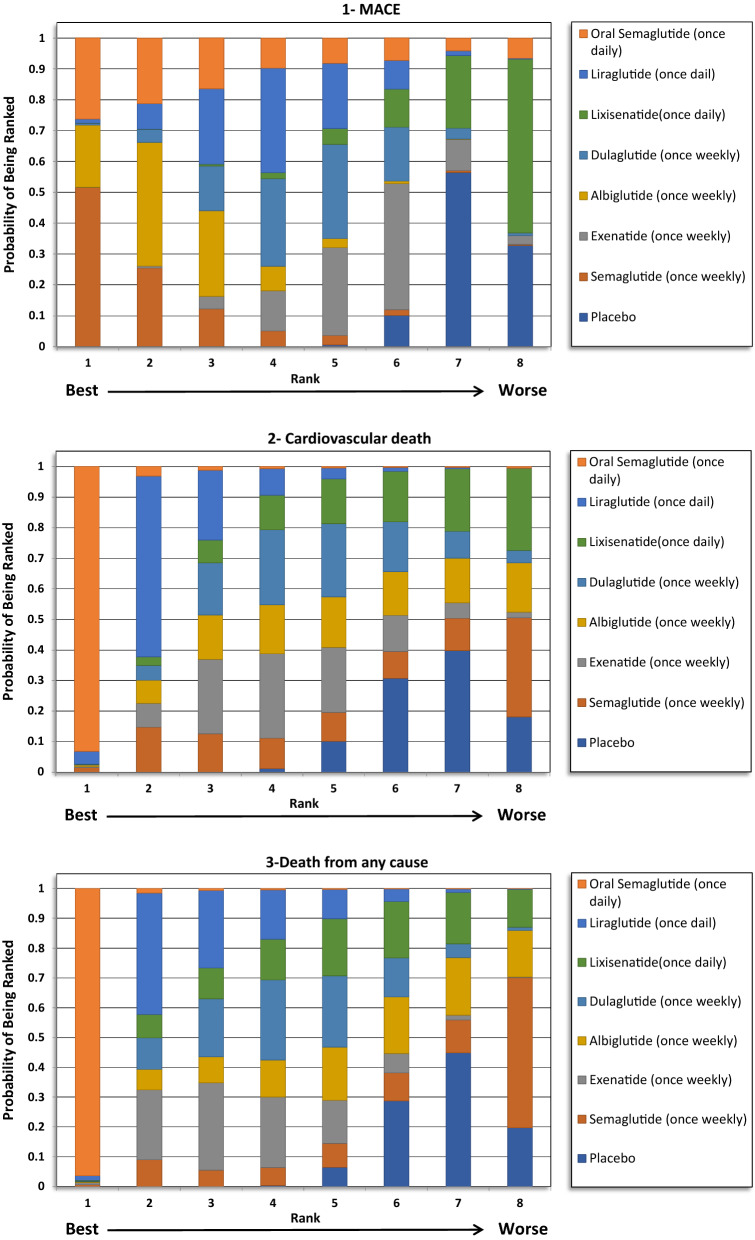

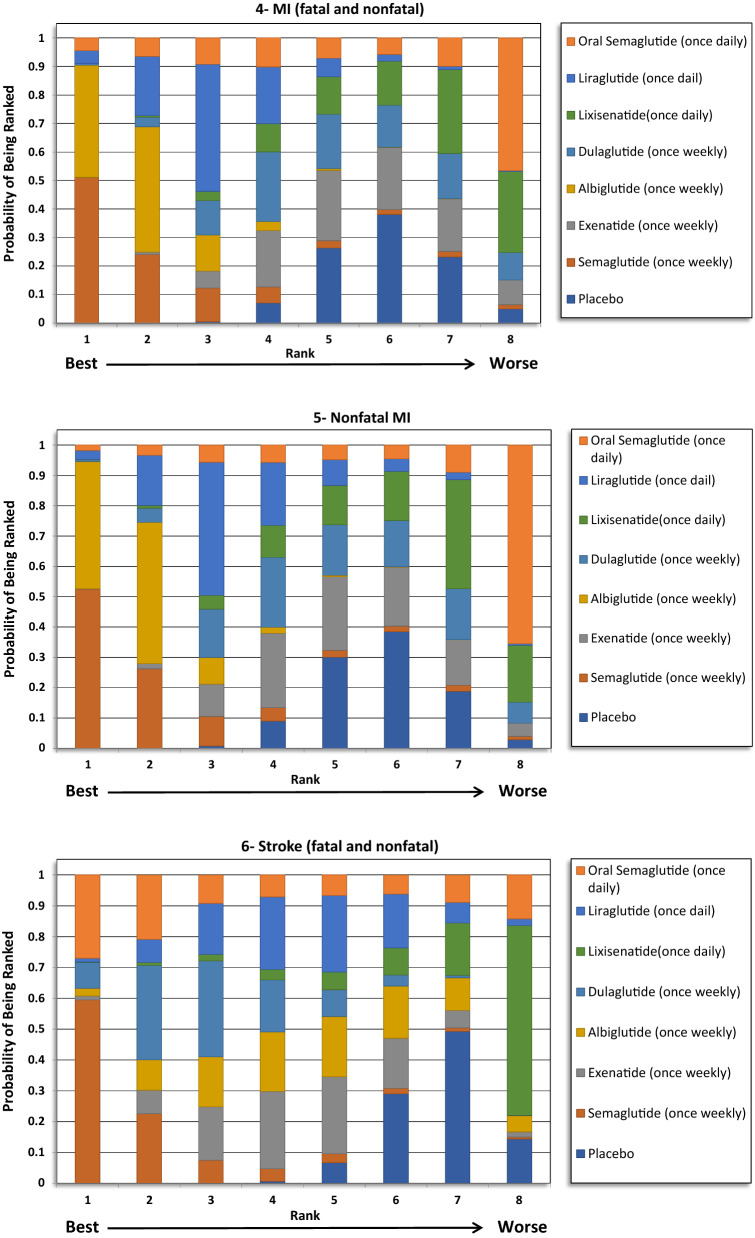

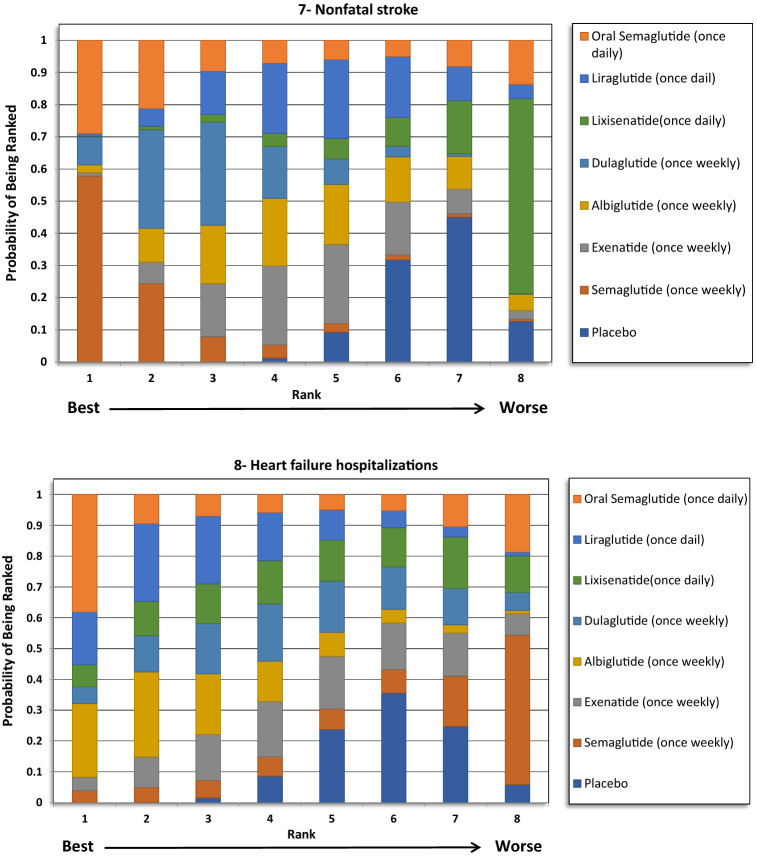
Ranking probability of GLP1RAs (Fixed Effects Rankogram)

The indirect comparison showed a statistically significant reduction in MACE following the use of weekly administered semaglutide compared to lixisenatide [odds ratio (OR) 0.71, 95% confidence intervals (CI) 0.52–0.96], and albiglutide compared to lixisenatide (OR 0.76, 95% CI 0.61–0.93). Moreover, the weekly injections of semaglutide, albiglutide, liraglutide (daily) and dulaglutide showed a statistically greater reduction in MACE when compared to placebo. The ranking results showed that weekly administration of semaglutide had the highest probability of being ranked first at 52% followed by oral semaglutide at 26% and albiglutide was ranked third at 20%. The indirect comparison of GLP1RAs is presented in Fig. 1, the ranking probability is presented in Fig. 2.

### CV death

The indirect comparison showed that oral semaglutide significantly reduced CV mortality compared with exenatide (OR 0.47, 95% CI 0.21–0.99), dulaglutide (OR 0.46, 95% CI 0.20–0.97), albiglutide (OR 0.45, 95% CI 0.19–0.97), and lixisenatide (OR 0.43, 95% CI 0.19–0.92). Moreover, oral semaglutide had the highest probability of being ranked first (> 90%) in reducing CV mortality in the rankogram comparison.

### Death from any cause

The indirect comparison showed that oral semaglutide significantly reduced death from any cause than all the GLP1RAs except liraglutide. Therefore, oral semaglutide had the highest probability of being ranked first at 96% in the rankogram comparison.

### Total MI (fatal and nonfatal)

No significant differences were detected regarding total MI between the treatments except when albiglutide was compared with exenatide and lixisenatide. Albiglutide was associated with a significant reduction in MI events compared to exenatide (OR 0.76, 95% CI 0.60–0.96) and lixisenatide (OR 0.72, 95% CI 0.55–0.93). The ranking results showed that weekly administration of semaglutide was ranked first at 51%, followed by albiglutide at approximately 40% in the rankogram comparison.

### Nonfatal MI

Similar to the results in total MI outcome, albiglutide was associated with a significant reduction in nonfatal MI compared to exenatide (OR 0.76, 95% CI 0.6–0.97) and lixisenatide (OR 0.72, 95% CI 0.55–0.93). However, weekly administration of semaglutide had the highest probability of being ranked first at approximately 52%, followed by albiglutide at 42%.

### Total stroke (fatal and nonfatal)

For total stroke events (fatal and nonfatal), no significant differences were identified between the treatments, except when the weekly administered semaglutide was compared to lixisenatide (OR 0.54, 95% CI 0.37–0.97). Moreover, ranking probability showed that weekly administered semaglutide was ranked first at 59%, followed by oral semaglutide at 27%.

### Nonfatal stroke

Similar to the results in total stroke outcome, no significant difference was identified between the GLP1RAs, except when the weekly semaglutide was compared to lixisenatide (OR 0.54, 95% CI 0.29–0.98). Furthermore, weekly semaglutide had the highest probability of being ranked first in reducing nonfatal stroke events at 58%, followed by oral semaglutide at 29%.

### Hospitalizations for HF

The indirect comparison did not show any significant difference among the GLP1RAs and placebo in reducing HF related hospitalizations. The ranking results showed that oral semaglutide had the highest probability of being ranked first, followed by albiglutide.

### Meta-regression analyses

The results from meta-regression analyses of the effect of potential covariates did not suggest any association between the covariates and the relative effect of GLP1RAs. The 95% CrI of the interaction coefficients for the included covariates crossed the null value (0). These findings were presented in Additional file 6: Table S3.

## Discussion

The study was conducted to indirectly compare the CV safety and mortality effects among different GLP1RAs in patients with T2DM using network meta-analysis (NMA), to identify the likely preferred agent with respect to CV safety. The study did not find any significant difference between GLP1RAs in reducing death from any cause, MI and stroke events. However, the ranking results showed that oral semaglutide had the highest probability to be ranked first (> 90%) in reducing CV death and death from any cause, while once weekly semaglutide had the highest probability to be ranked first in reducing MI and stroke events.

Several meta-analyses were performed to compare the CV safety of GLP1RAs. Furthermore, NMA was also performed to explore the CV safety of GLP1RAs in comparison with other hypoglycemic drugs such as SGLT2is [24–26]. Five meta-analyses were conducted including data from all seven GLP1RAs CVOTs published to date, Kristensen et al. [15], Giugliano et al. [16], Mannucci et al. [17], Marsico et al. [18], and Zhu et al. [19]. The results from these five meta-analyses were summarized in Additional file 7: Table S4. In the Kristensen et al. meta-analysis, GLP1RAs was associated with a 12% reduction in the three-point MACE (hazard ratio (HR) 0.88, 95% CI 0.82–0.94) with moderate heterogeneity reported [15]; the results from the other four meta-analyses were very similar to that of Kristensen et al. [16–19]. Prior to the addition of REWIND and PIONEER-6 trials data, the reduction ranged between 10% (HR 0.90, 95% CI 0.82–0.99) and 13% (OR 0.87, 95% CI 0.82–0.93) as reported by Bethel et al. and Fei et al. meta-analyses [27, 28]. The subgroup analysis in the meta-analysis by Kristensen et al. showed that the reduction in MACE associated with GLP1RAs was regardless of the median follow-up of the studies, body mass index, and HbA1c at baseline. However, no significant reduction in MACE was seen with daily medications, patients with no established CVD (primary prevention), or with exendin-4 based GLP1RAs. When considering the individual CVOTs, MACE was significantly reduced in the LEADER (liraglutide) [8], SUSTAIN-6 (semaglutide once weekly) [9], Harmony outcomes (albiglutide) [11], and REWIND (dulaglutide) trials [12]. In the current NMA, the indirect comparisons did not show any significant differences between these drugs when indirectly compared to each other with respect to the MACE outcome. However, weekly administration of semaglutide was associated with a 29% reduction in MACE events compared to lixisenatide (OR 0.71, 95% CI 0.52–0.96), and albiglutide was associated with a 24% reduction in MACE events compared to lixisenatide (OR 0.76, 95% CI 0.61–0.93). These results have the potential to support the case that human based GLP1RAs are more likely to be associated with a significant reduction in MACE compared to exendin-4 based GLP1RAs. However, it is important to note that, in the subgroup analysis previously reported by Kristensen et al. the exendin-4 based drugs showed significant heterogeneity, which might suggest the existence of differences in the trial’s populations and design, more than the chemical structure. Nevertheless, in our analysis, the weekly semaglutide was the preferred agent among all of the GLP1RAs based on ranking probability results.

When examining the individual components of MACE, a significant reduction in CV mortality (12–13%) and stroke (16–17%) was consistently reported for GLP1RAs, with no heterogeneity, in the five aforementioned meta-analyses. However, the reduction in the MI events (8–9%) was not as robust as the results for the CV mortality or the stroke outcomes, as this was only significant in three of the five meta-analyses [15–19]. Moreover, the reduction in MI events was significant in two CVOTs, namely the LEADER (liraglutide) and Harmony outcomes (albiglutide) trials. However, the remaining trials did not show a significant reduction. The CV mortality result was largely driven by the significant reduction that was seen in the LEADER (liraglutide) and PIONEER-6 (oral semaglutide) trials. Avgerinos et al. found oral semaglutide to be superior to placebo in term of CV mortality (OR 0.55, 95% CI 0.31–0.98), but that was not significant when oral semaglutide was compared to other antidiabetic agents, including liraglutide [29]. In our analysis, the indirect comparison showed that oral semaglutide was associated with a greater reduction in CV mortality events compared with exenatide, lixisenatide, albiglutide and dulaglutide, but not when compared with liraglutide or once weekly semaglutide. However, the ranking results showed that oral semaglutide was the preferred GLP1RA by more than 90% probability. Furthermore, the indirect comparison did not provide significant differences in relation to MI and stroke reduction between the GLP1RAs. Although, weekly semaglutide had the highest probability of being ranked first for both outcomes.

Death from any cause was also reported to be significantly reduced by GLP1RAs. The reduction ranged between 10 and 12% in four of the five meta-analyses, with no significant heterogeneity reported [15–18], and the last meta-analysis did not evaluate this outcome [19]. Such reduction was largely driven by the PIONEER-6 (oral semaglutide) trial, and that was previously reflected in the Avgerinos et al. meta-analysis when they compared oral semaglutide to placebo (OR 0.58, 95% CI 0.37–0.92) [29]. In our analysis, oral semaglutide significantly reduced the deaths from any cause when indirectly compared with all other GLP1RAs, except liraglutide which was also reported by the Avgerinos et al. meta-analysis [29]. Moreover, oral semaglutide was ranked first in comparison with other GLP1RAs with a probability of more than 90%. This result is very promising for the first oral GLP1RAs. Although, it cannot be entirely explained given the lack of heterogeneity. PIONEER-6 was the shortest trial in terms of duration and included patients with a longer duration of T2DM at baseline (14.9 years) and had the highest use of SGLT2is at 9%. Although CV mortality and all-cause mortality events were significantly reduced in the PIONEER-6 trial, the reduction in MACE did not reach statistical significance [13].

For the HF related hospitalization, the use of SGLT2is was previously found to be associated with the most cardioprotective effect in patients with HF, or even at risk of having HF, compared to all other antidiabetic classes, including GLP1RA [28, 30]. However, unlike previously reported meta-analyses, Kristensen et al., Giugliano et al., and Fei et al. were the first to report a significant reduction in the rate of HF related hospitalizations, and later on Marsico et al. and Zhu et al. reported the same finding in their most recent meta-analyses. GLP1RAs were associated with 8–13% reduction in the rate of HF related hospitalizations, with no heterogeneity [15, 16, 18, 19, 28]. The Harmony outcomes (albiglutide) trial was the only trial to show statistical significance regarding reduction in HF related hospitalizations. In our analysis, no significant difference was found among the GLP1RAs, including albiglutide. However, it is important to note that albiglutide is no longer available in the market, as per the decision made by GlaxoSmithKline in 2017 [31]. Regardless of the modest clinical significance of this result to patients with established or at risk of HF, knowing that GLP1RAs may have a modest positive effect is very assuring for the use of GLP1RAs for combined drug therapy with SGLT2is or as an alternative to SGLT2is in patients with T2DM and established or at risk of HF. The Fei et al. meta-analysis [28] indicated the superiority of GLP1RAs over dipeptidyl peptidase-4 inhibitors (DPP-4i) in term of HF related hospitalization, this finding negated data from an observational cohort study that was previously conducted by Dawwas et al. [32] using real world data. Therefore, more research is needed to assess the superiority of GLP1RAs and SGLT2is over other classes of antidiabetic agents in real-world practice.

The risk of CVD, CV mortality, and death from any cause varies among patients with T2DM based on age, HbA1c level, history of CVD, and the duration of diabetes [33–35]. The use of GLP1RA, such as once weekly semaglutide in the SUSTAIN 6 trial, was found to be an independent predictor for the reduction in the rate of MACE, CV mortality, nonfatal MI, and nonfatal stroke [36]. This was supported by the findings from the meta-regression in the current study, as all the CV benefits were independent of age, duration of diabetes, mean HbA1c level, and the existence of CVD at baseline. This indicates that the CV protective effect for GLP1RA can be seen in most patients with T2DM; therefore, all patients that have established or at high risk of CVD would benefit from being initiated on GLP1RA regardless of their age, duration of diabetes, and HbA1c level. Moreover, when using one of the GLP1RAs, the addition of another agent with CV protective effect, such as one of the SGLT2i, can contribute to even better CV outcomes in these patients [37]. Therefore, in patients with established or at high risk of CVD when additional therapy is needed for better control of diabetes, agents with CV protective effect should be considered.

In light of the recent results of CVOTs, the diabetes guidelines have changed their recommendations regarding T2DM treatment. This change was heavily influenced by the CV effect of drugs regardless of the glycemic effect. The most recent American Diabetes Association (ADA) guidelines of 2020 recommend the use of GLP1RAs as an add-on to metformin or as a first line therapy for selected patients who cannot tolerate metformin [3]. Moreover, the use of the long-acting GLP1RAs is recommended prior to the addition of basal insulin. For patients with established or at high risk of CVD, administration of GLP1RAs and SGLT2is—that have demonstrated CVD benefit—should be undertaken regardless of glycemic control. For those with HF (especially with reduced ejection fraction), the use of SGLT2is is preferred over other drug classes since canagliflozin, dapagliflozin, and empagliflozin demonstrated HF benefit. The ADA guidelines recommend the use of GLP1RAs as an alternative to those who cannot tolerate SGLT2is [3]. This recommendation is consistent with the recent meta-analyses that has shown a statistically significant reduction in HF hospitalizations with GLP1RAs [15, 16, 18, 19, 28].

### Limitations

In this paper, other safety issues were not addressed, such as retinopathy. However, recent meta-analysis did not show significant increase in retinopathy events with the use of GLP1RAs [15]. There are differences in terms of populations among the trials that cannot be controlled by the NMA design. However, we explored key variables in the baseline characteristics of patients included in the trials using the meta-regression methods that did not suggest major variations between these populations. Moreover, the included trials used a composite primary endpoint (MACE) to achieve sufficient power but some components of MACE that drive the event number may differ from study to another. Therefore, the results should be interpreted cautiously. For stroke events, some trials reported either nonfatal events only or total stroke events only. Although albiglutide is no longer available in the market, given that albiglutide was included in previously reported meta-analyses, albiglutide data was included in this NMA. Given that the researchers are very familiar with the most recent work that were done and published in this field, an extensive literature search on multiple databases was not deemed necessary, and therefore was not conducted. Lastly, when relying on the findings from this research the reader should acknowledge that this study did not focus on some other important decision-making parameters, such as cost of medications, weight reduction, glycemic control and other safety issues. Therefore, these aspects would need to be reviewed from other research, and if this information are not available then it would be an area for future research.

## Conclusion

The GLP1RAs have shown significant benefits in terms of CV safety. While utilizing data from the CVOTs to date, the indirect comparison and ranking probability results have shown that one weekly semaglutide and oral semaglutide seems to be the preferred option in patients with T2DM and established or at high risk of CVD. This result can aid health care providers, pharmacies and therapeutics committees in hospitals, and insurance companies when deciding which GLP1RA to start or add to their formulary.

## Supplementary information

**Additional file 1: Table S1.** PRISMA Network Meta-Analysis Checklist.

**Additional file 2: Figure S1.** Flow diagram for study selection.

**Additional file 3: Figure S2.** Network plot of all interventions in the analysis.

**Additional file 4: Table S2.** Risk of bias assessment of glucagon like peptide-1 receptor agonist (GLP1RA) cardiovascular outcome trials (CVOTs).

**Additional file 5: Figure S3.** Inconsistency plots for the outcomes from the fixed effect model.

**Additional file 6: Table S3.** Results from the meta-regression analyses for the interaction coefficient, median (95% CrI).

**Additional file 7: Table S4.** Summary of the results from the previous pairwise meta-analyses.

## Data Availability

All data generated or analyzed during this study are included in this published article [and its additional information files].

## Abbreviations

CV: Cardiovascular
CVD: Cardiovascular disease
GLP1RA: Glucagon like peptide-1 receptor agonist
T2DM: Type 2 diabetes mellitus
CVOT: Cardiovascular outcome trial
NMA: Network meta-analysis
MI: Myocardial infarction
MACE: Major adverse cardiovascular events
HF: Heart failure
SGLT2i: Sodium glucose transporter-2 inhibitor
