# Supplementary material for: Indirect comparison of glucagon like peptide-1 receptor agonists regarding cardiovascular safety and mortality in patients with type 2 diabetes mellitus: network meta-analysis
Source: Cardiovasc Diabetol. 2020 Jun 22;19:96. doi: 10.1186/s12933-020-01070-z (PMC7310317; doi:10.1186/s12933-020-01070-z)
Supplement: Supplementary file 2 — Additional file 2: Figure S1. Flow diagram for study selection. [file 12933_2020_1070_MOESM2_ESM.docx]

Potentially relevant studies identified and screened for retrieval

(n = 79)

Studies excluded based on title and abstract screening (n = 69)

Studies retrieved for further evaluation (n = 10)

Studies excluded after full article screening:

Methodology and baseline characteristics of trials

(n = 3)

RCTs included in the systematic review and network meta-analysis

(n = 7)

Figure S1. Flow diagram for study selection
