# Supplementary material for: Indirect comparison of glucagon like peptide-1 receptor agonists regarding cardiovascular safety and mortality in patients with type 2 diabetes mellitus: network meta-analysis
Source: Cardiovasc Diabetol. 2020 Jun 22;19:96. doi: 10.1186/s12933-020-01070-z (PMC7310317; doi:10.1186/s12933-020-01070-z)
Supplement: Supplementary file 3 — Additional file 3: Figure S2. Network plot of all interventions in the analysis. [file 12933_2020_1070_MOESM3_ESM.docx]

|  |
| --- |
| Figure S2. Network plot of all interventions in the analysis |
