# Supplementary material for: Indirect comparison of glucagon like peptide-1 receptor agonists regarding cardiovascular safety and mortality in patients with type 2 diabetes mellitus: network meta-analysis
Source: Cardiovasc Diabetol. 2020 Jun 22;19:96. doi: 10.1186/s12933-020-01070-z (PMC7310317; doi:10.1186/s12933-020-01070-z)
Supplement: Supplementary file 4 — Additional file 4: Table S2. Risk of bias assessment of glucagon like peptide-1 receptor agonist (GLP1RA) cardiovascular outcome trials (CVOTs). [file 12933_2020_1070_MOESM4_ESM.docx]

| Table S2. Risk of bias assessment of glucagon like peptide-1 receptor agonist (GLP1RA) cardiovascular outcome trials (CVOTs) | | | | | | | | |
| --- | --- | --- | --- | --- | --- | --- | --- | --- |
| **Study** | **Publication Year** | **Selection Bias** | | **Performance Bias** | **Detection Bias** | **Attrition Bias** | **Reporting Bias** | **Other Bias** |
|  |  | Random sequence generation | Allocation concealment | Blinding of participants and personnel | Blinding of outcome assessment | Incomplete outcome data | Selective reporting | Other bias |
| **ELIXA** | 2015 | Low | Low | Low | Low | Low | Low | Unclear |
| **LEADER** | 2016 | Low | Low | Low | Low | Low | Low | Unclear |
| **SUSTAIN-6** | 2016 | Low | Low | Low | Low | Low | Low | Unclear |
| **EXSCEL** | 2017 | Low | Low | Low | Low | Low | Low | Unclear |
| **HARMONY** | 2018 | Low | Low | Low | Low | Low | Low | Unclear |
| **REWIND** | 2019 | Low | Low | Low | Low | Low | Low | Unclear |
| **PIONEER-6** | 2019 | Low | Low | Low | Low | Low | Low | Unclear |
| Each domain of risk was assigned “Low” for low risk, “High” for high risk, and “Unclear” for unclear risk.  Abbreviations: ELIXA: the evaluation of lixisenatide in acute coronary syndrome trial. LEADER: the liraglutide effect and action in diabetes evaluation of cardiovascular outcome results trial. SUSTAIN-6: the preapproval trial to evaluate cardiovascular and other long-term outcomes with semaglutide in subjects with type 2 diabetes. EXSCEL: the exenatide study of cardiovascular event lowering. HARMONY: a long term, randomized, double blind, placebo-controlled study to determine the effect of albiglutide, when added to standard blood glucose lowering therapies, on major cardiovascular events in patients with type 2 diabetes mellitus. | | | | | | | | |
