# Supplementary material for: Indirect comparison of glucagon like peptide-1 receptor agonists regarding cardiovascular safety and mortality in patients with type 2 diabetes mellitus: network meta-analysis
Source: Cardiovasc Diabetol. 2020 Jun 22;19:96. doi: 10.1186/s12933-020-01070-z (PMC7310317; doi:10.1186/s12933-020-01070-z)
Supplement: Supplementary file 5 — Additional file 5: Figure S3. Inconsistency plots for the outcomes from the fixed effect model. [file 12933_2020_1070_MOESM5_ESM.docx]

Figure S3. Inconsistency plots for the outcomes from the fixed effect model

1- MACE

2- CV death

3- Death from any cause

4- Total MI (fatal and nonfatal)

5- Nonfatal MI

6- Total stroke (fatal and nonfatal)

7- Nonfatal stroke

8- Hospitalizations for heart failure (HF)
