# Supplementary material for: Indirect comparison of glucagon like peptide-1 receptor agonists regarding cardiovascular safety and mortality in patients with type 2 diabetes mellitus: network meta-analysis
Source: Cardiovasc Diabetol. 2020 Jun 22;19:96. doi: 10.1186/s12933-020-01070-z (PMC7310317; doi:10.1186/s12933-020-01070-z)
Supplement: Supplementary file 6 — Additional file 6: Table S3. Results from the meta-regression analyses for the interaction coefficient, median (95% CrI). [file 12933_2020_1070_MOESM6_ESM.docx]

| Table S3. Results from the meta-regression analyses for the interaction coefficient, median (95% CrI) | | | | | | |
| --- | --- | --- | --- | --- | --- | --- |
| **Outcome** | **Age** | **Median follow-up**  **Years** | **Duration of diabetes**  **years** | **HbA1c**  **Mean %** | **Existence of CVD %** | **Statins Use**  **N (%)** |
| MACE | -0.001 (-3.46, 3.41)^*^ | -0.003 (-2.25, 2.01) | -0.06 (-1.63, 1.75) | 0.05 (-2.12, 7.26) | 0.004 (-2.88, 10.63) | -0.02 (-3.67, 2.58) |
| CV death | -0.04 (-32.72, 10.17) | 0.01 (-5.76, 11.73) | 0.06 (-4.59, 7.11) | -0.27 (-25.50, 3.88) | 0.03 (-6.10, 7.15) | -0.02 (-4.95, 4.24) |
| Death from any cause | 0.10 (-5.71, 142.63) | -0.01 (-4.89, 3.35) | 0.01 (-5.25, 4.69) | 0.01 (-2.88, 3.78) | 0.03 (-5.59, 17.41) | 0.05 (-4.35, 6.74) |
| MI | 0.00 (-4.03, 3.57) | 0.01 (-1.74, 2.26) | -0.03 (-2.57, 1.39) | -0.01 (-3.55, 2.46) | 0.00 (-3.21, 2.75) | -0.01 (-7.37, 4.06) |
| Non-fatal MI | 0.00 (-3.58, 3.75) | 0.00 (-2.27, 1.94) | 0.00 (-1.76, 2.07) | 0.01 (-2.89, 4.49) | 0.00 (-3.39, 2.82) | 0.00 (-1.84, 2.51) |
| Stroke | 0.01 (-5.85, 13.65) | -0.03 (-6.20, 2.70) | -0.01 (-6.71, 3.57) | 0.02 (-2.94, 3.27) | -0.01 (-5.50, 4.31) | 0.03 (-2.40, 4.68) |
| Non-fatal stroke | -0.03 (-13.25, 5.16) | 0.00 (-4.30, 3.54) | -0.02 (-3.59, 2.45) | 0.03 (-2.44, 3.99) | 0.01 (-3.61, 5.24) | 0.00 (-3.10, 3.03) |
| Hospitalization due to heart failure | 0.00 (-1.79, 1.63) | -0.01 (-1.42, 1.02) | 0.01 (-1.20, 2.11) | 0.00 (-2.29, 1.92) | 0.001 (-1.64, 1.86) | 0.00 (-1.07, 1.56) |
| ^*^ The interaction coefficient: median (95%CrI) | | | | | | |
