# Supplementary material for: Indirect comparison of glucagon like peptide-1 receptor agonists regarding cardiovascular safety and mortality in patients with type 2 diabetes mellitus: network meta-analysis
Source: Cardiovasc Diabetol. 2020 Jun 22;19:96. doi: 10.1186/s12933-020-01070-z (PMC7310317; doi:10.1186/s12933-020-01070-z)
Supplement: Supplementary file 7 — Additional file 7: Table S4. Summary of the results from the previous pairwise meta-analyses. [file 12933_2020_1070_MOESM7_ESM.docx]

| Table S4. Summary of the results from the previous pairwise meta-analyses | | | | | | | | |
| --- | --- | --- | --- | --- | --- | --- | --- | --- |
| Author of the  Meta-analysis | MACE | CV  Mortality | Nonfatal  MI | Nonfatal stroke | MI | stroke | All-cause Mortality | Hospitalization for HF |
| Kristensen et al [15] | **0.88**  **(0.82-0.94)** | **0.88**  **(0.81–0.96)** | NR | NR | **0.91**  **(0.84-1.00)** | **0.84**  **(0.76-0.93)** | **0.88**  **(0.83-0.95)** | **0.91**  **(0.83-0.99)** |
| Giugliano et al [16] | **0.87**  **(0.80-0.96)** | **0.88**  **(0.78-0.98)** | 0.92  (0.81-1.04) | **0.84**  **(0.75-0.99)** | NR | NR | **0.89**  **(0.79-0.99)** | **0.91**  **(0.86-0.97)** |
| Mannucci et al [17] | **0.87**  **(0.81-0.93)** | **0.88**  **(0.80-0.97)** | 0.90  (0.8-1.0) | **0.83**  **(0.75-0.93)** | **0.91**  **(0.83-0.99)** | **0.83**  **(0.75-0.91)** | **0.90**  **(0.82-0.98)** | 0.93  (0.85-1.02) |
| Marsico et al [18] | **0.88**  **(0.80-0.96)** | **0.88**  **(0.79–0.98)** | NR | NR | 0.91  (0.82-1.02) | **0.84**  **(0.76-0.94)** | **0.89**  **(0.81-0.97)** | **0.92**  **(0.86-0.97)** |
| Zhu et al [19] | **0.88**  **0.84-0.92)** | **0.87**  **0.81-0.94** | NR | NR | **0.92**  **0.86-0.99** | **0.84**  **0.77-0.93** | NR | **0.90**  **0.83-0.99** |
| All data represented were hazard ratio with 95% confidence interval, except for Mannucci et al [17] which was Mantel–Haenszel odds ratio with 95% confidence interval, and Zhu et al [19] which was relative risk with 95% confidence interval. Bold numbers represent significant outcomes.  MACE: major adverse cardiovascular events; CV: cardiovascular; MI: myocardial infarction; HF; heart failure; NR: not reported | | | | | | | | |
